# Forced displacement: critical lessons in the protracted aftermath of a flood disaster

**DOI:** 10.1007/s10708-021-10471-w

**Published:** 2021-07-15

**Authors:** Blessing Mucherera, Samuel Spiegel

**Affiliations:** 1 School of GeoSciences, The University of Edinburgh, Drummond St, Edinburgh, EH8 9XP UK; 2 Centre of African Studies School of Social and Political Science, University of Edinburgh, Chrystal Macmillan Building, 15a George Square, Edinburgh, EH8 9LD UK

**Keywords:** Flood-induced resettlement, Displacement, Power, political ecology, Disaster

## Abstract

Forced displacement and resettlement is a pervasive challenge being contemplated across the social sciences. Scholarly literature, however, often fails to engage complexities of power in understanding socio-environmental interactions in resettlement processes. Addressing Zimbabwe’s Tokwe-Mukosi flood disaster resettlement, we explore hegemonic uses of state power during the pre- and post-flood induced resettlement processes. We examine how state power exercised through local government, financial, and security institutions impacts community vulnerabilities during forced resettlement processes, while furthering capitalist agendas, drawing insights from analysing narratives between 2010 and 2021. Concerns abound that multiple ministries, the police, and the army undermined displaced people’s resilience, including through inadequate compensation, with state institutions neglecting displaced communities during encampment by inadequately meeting physical security, health, educational, and livestock production needs. We explore how forcibly resettling encamped households to a disputed location is not only an ongoing perceived injustice regionally but also a continuing reference point in resettlement discussions countrywide, reflecting concerns that land use and economic reconfigurations in resettlement can undermine subsistence livelihoods while privileging certain values and interests over others. Policy lessons highlight the need for reviewing disaster management legislation, developing compensation guidelines and reviewing encampment practices. Analytically, lessons point to how state power may be studied in relation to perspectives on the destruction of flood survivors’ connections to place, people and livelihoods, underscoring the critical need for theorising the relationships between power dynamics and diverse experiences around displacement.

## Introduction

Globally, the disproportionate impacts of flood-induced displacement, predominantly on low-income and low-lying households, are raising pressing concerns for resettling internally displaced communities (United Nations Office for Disaster Risk Reduction [UNDRR], 2019). ‘Resettlement’—a complex and multi-faceted phenomenon frequently used by governments to permanently move communities from perceived dangerous situations to new locations (Mortreux et al., 2018)—has recently resurged to prominence as a point of debate (Ferris, 2014; McMichael et al., 2019), particularly where government infrastructure and development projects led to diverse social, economic and political inequities (Hino et al., 2017; McNamara et al., 2018; Weir & Pittock, 2017). In response to observations that forced resettlement is still understudied empirically and under-theorised (McMichael et al. (2019), we draw on resettlement studies and critical political ecology fields, to explore resettlement challenges in Zimbabwe, where disasters such as cyclones and floods have, in recent years, layered onto ongoing political-economic instability.

In this study we focus on the 2014 Tokwe-Mukosi flood disaster which displaced close to 50,000 villagers (Chitimira, 2017). Tokwe-Mukosi is Zimbabwe’s first major post-independence dam-induced displacement in which climate stresses contributed significantly to both the immediate time of the disaster and the subsequent struggles. While not ignoring the struggles of communities resettled before the Tokwe-Mukosi flood disaster, we focus more on the population affected by the 2014 floods. As the first major disaster to displace such a significant number of people in Zimbabwe, it set a precedent for future resettlements and remains a contentious reference point for discussing a broader constellation of disasters in Zimbabwe. Building on insights from the first author’s visit to the Tokwe-Mukosi area in 2014 and ethnographic research with flood disaster survivors in other regions of Zimbabwe over the last several years by the second author, we critically analyse literature from 2010 to 2021 to probe evolving themes on flood and dam-induced resettlement and power dynamics centred on the struggles that occurred in Tokwe-Mukosi. Studies on the Tokwe-Mukosi disaster have focused on human rights (Chitimira, 2017; Hove, 2016), policy (Chipangura et al., 2019), vulnerability (Mavhura et al., 2017; Mukwashi, 2017; Mutangi & Mutari, 2014), livelihoods (Chazireni & Chigonda, 2018), stakeholder coordination (Zikhali, 2018), and traditional leadership (Tarisayi, 2018) perspectives. Yet, few scholarly sources have explored how state power influenced vulnerabilities and undermined resilience building in the Tokwe-Mukosi flood-disaster resettlement processes. We therefore examine how the state–using the Ministry of Finance and Economic Development (MFED), Ministry of Local Government and Public Works (MLGPW), the Zimbabwe Republic Policy (ZRP) and the Zimbabwe National Army (ZNA) to control the compensation processes–encamped internally displaced persons (IDPs) in a transit camp, and eventually forced them to settle in a disputed Chingwizi site, thereby exacerbating their vulnerability. The overall aim of the study is to explore the impact of the hegemonic uses of state power in pre- and post-flood induced resettlement processes, using the 2014 Tokwe-Mukosi flood disaster as a case study.

We draw on discussions in political ecology scholarship which offers analytical tools for moving beyond simplifying institutional rhetoric about resilience to explore how various faces of state power can serve capitalist interests that increase environmental vulnerability (Arnall, 2014; Cote & Nightingale, 2012). The following research questions drive this study: (i) What processes characterised the Tokwe-Mukosi pre- and post-flood induced resettlement period? (ii) How did state power contribute to the vulnerability of the Tokwe-Mukosi communities’ forced resettlement? And (iii) Which lessons from the Tokwe-Mukosi resettlement process have been or have not been acted upon? We introduce our political ecology analysis in the next section, identifying gaps in flood-induced resettlement literature that beckon this analytic turn. The third section then introduces the Tokwe-Mukosi resettlement context along with our methodological approach. The fourth section discusses the results of our analysis before moving to the fifth and final section, where we draw out some critical implications, integrating our observations from Cyclone Idai in 2019 and Chilonga displacements in 2021.

## Contextualising “resettlement”: experiences, institutional shortcomings and political ecology

Literature on resettlement has embraced various, at times countervailing, tendencies. John et al. (2019) and Correa (2011) argue that preventive resettlement should be carried out as a last resort when flood hazards are uncontrollable and of high risk. Such pre-emptive measures reduce the exposure of vulnerable groups and their assets to flood risk by physically removing them from the threatened location to a safer location (Klepp & Herbeck, 2016; Mortreux et al., 2018). McMichael et al. (2019), for example, describe how villages in Fiji were assisted by the government to resettle when risks of flooding reached intolerable levels. However, resettlement can also be tied to other non-lifesaving priorities such as political legitimacy and identity. In India, Ghoramara and Lohachara, where scientists expect climate change to intensify land losses due to sea-level rise (Nandi et al., 2016), resettlement coincided with the ruling party’s desire to demonstrate a commitment to social welfare and resettlement in a habitable location (Mortreux et al., 2018). On the other hand, in the Pacific Islands, Farbotko et al. (2016) observed that some Tuvaluans challenged forced resettlement, risking drowning in order to preserve their identities and homes. Conversely, Albert et al. (2018) noted that in Alaska, indigenous communities in Shishmaref are yet to be relocated since 2002 due to limited government commitment to fund essential services, infrastructure, and housing in the new site. The point here is that the literature is replete with stories illustrating mismatches of the priorities of local communities and those of state actors.

International frameworks, such as the Sendai Framework for Disaster Risk Reduction 2015–2020 (SFDRR), are meant to encourage the development of public policies from the national down to the local level to address relocations of communities in disaster-prone areas (United Nations International Strategy for Disaster Reduction [UNISDR], 2015). Countries such as Fiji (Republic of Fiji, 2017) and Kiribati (Klepp & Herbeck, 2016), for example, have developed guidelines to support proactive resettlement, and John et al. (2019) argue for the strengthening of governing institutions responsible for implementing such plans. However, as noted by others (van Niekerk et al., 2020) implementing policies with targets measured against international frameworks such as the SFDRR require understanding the contextual historical and socioeconomic environment that influences the outcomes.

Critiques of camps used to accommodate victims of forced displacements have been ongoing for at least close to half a century (Chambers, 1979; Hovil, 2007; Schmidt, 2003). While governments still use camps as the central point for offering emergency aid services and security, these complex spaces are also hubs of increased vulnerability due to inadequate access to food, water, shelter, health care and other non-food items by IDPs (Ekezie et al., 2019; Singh et al., 2016) as well as restricted livelihood opportunities (Ekezie et al., 2019) particularly when state institutions curtail movements, access to resources, ownership of assets, and employment options for displaced communities (Bakewell, 2014). Additionally, these communities are sometimes relocated far away from their original location, which affects their ability to immediately embark on productive livelihoods (Arnall et al., 2013; Tadgell et al., 2018).

As it is the responsibility of the affected nation’s government to sustain its citizens (Ahmad, 2017), humanitarian assistance to IDPs by international organisations is less established compared to refugee settings (United Nations, 2004), and governments in Tanzania, Zambia, Sudan, and Uganda seem to have provided displaced communities with land without encamping them (Bakewell, 2014). Nonetheless, site selection is a critical aspect of the resettlement process, ideally achieved through giving communities the right to choose where they want to resettle (Nygren & Wayessa, 2018) equipped with knowledge on the level of hazard exposure which they perceive to be manageable. Reckless and haphazard resettlement of communities places them in the same or more dangerous conditions than they were before. John et al. (2019) observed that in Tanzania, the Mabwepande community resettled by the government due to floods, found themselves in an area with massive soil erosion actually increasing the risk of the floods inundating some of the houses.

Governments have also used diverse strategies of compensating resettled IDPs, most commonly cash payments and land (Tadgell et al., 2018). Some governments prefer cash compensation as it is logistically more straightforward and faster to allocate than land (Rowan, 2017). Also, Mariotti (2014) observes that IDPs often prefer cash compensation because it is immediate, which reduces the risks of governments failing to fulfil their promised follow-up support; the Gbagye tribe, which was displaced by the Nigerian government in the late 1970s to allow the construction of Abuja, the federal capital, has yet to be compensated (Akume, 2015). Nonetheless, the value of the cash compensation is not able to compensate for intangible losses such as social networks, income and culture (Al Atahar, 2014; Roca & Villares, 2012).

To optimise cultural preservation and social networks, López-Carr and Marter-Kenyon (2015) suggest the relocation of an entire village together; the Vunidogoloa villages in Fiji maintained the same spatial configuration when they resettled (McMichael et al. (2019). However, agricultural livelihoods might be under threat in the new location (Al Atahar, 2014), such that some scholars suggest the need for developing new industries apart from agriculture and complemented by both new and old skills training with a long-term focus (Usamah & Haynes, 2012). The point here is that displacement experiences and needs after disasters are varied.

Miller (2020: 1572) defines just resilience ‘as the conditions that enable people to cope with, recover and restore their livelihoods in fair, equitable and inclusive ways following shocks and disturbances, such as displacement, while also maintaining essential and valued connections to place, community and economy.’ We argue that in studying resettlement, it is essential to dissect how power dynamics associated with state institutions influence resilience post disaster and that integrating concepts of power allows for a clearer understanding of divergent choices and inequalities. Exploring various injustices in the resettlement planning processes, while highlighting power imbalances that scholars and practitioners need to address, this study thus aims to apply a political ecology analysis to understand forced resettlement following a dam failure in a context of climate change exacerbations. Political ecology offers avenues for de-stabilising homogenising assumptions about policy discourses and marginalisation, giving attention to diverse histories, changing discourses and different social circumstances, raising questions about whose ‘context’ matters and how particular forms of hegemony and power are experienced at given points in time (Arnall, 2014; Benjaminsen & Svarstad, 2021; Spiegel, 2017; Sultana, 2020). With a political ecology analysis of what has been termed a society-nature interface (Ingalls & Stedman, 2016), we aim to add to the emerging body of literature in this regard (Boonstra, 2016; Chandler, 2014; Cote & Nightingale, 2012; Cretney, 2014; Evans & Reid, 2014; Fabinyi et al., 2014; and Ingalls & Stedman, 2016). Our case study focuses on how state actors in Zimbabwe’s Ministry of Finance and Economic Development, Ministry of Local Government and Public Works, courts, police, and army influenced the resettlement processes in Tokwe-Mukosi.

## Context and methodological approach

**Fig. 1:**
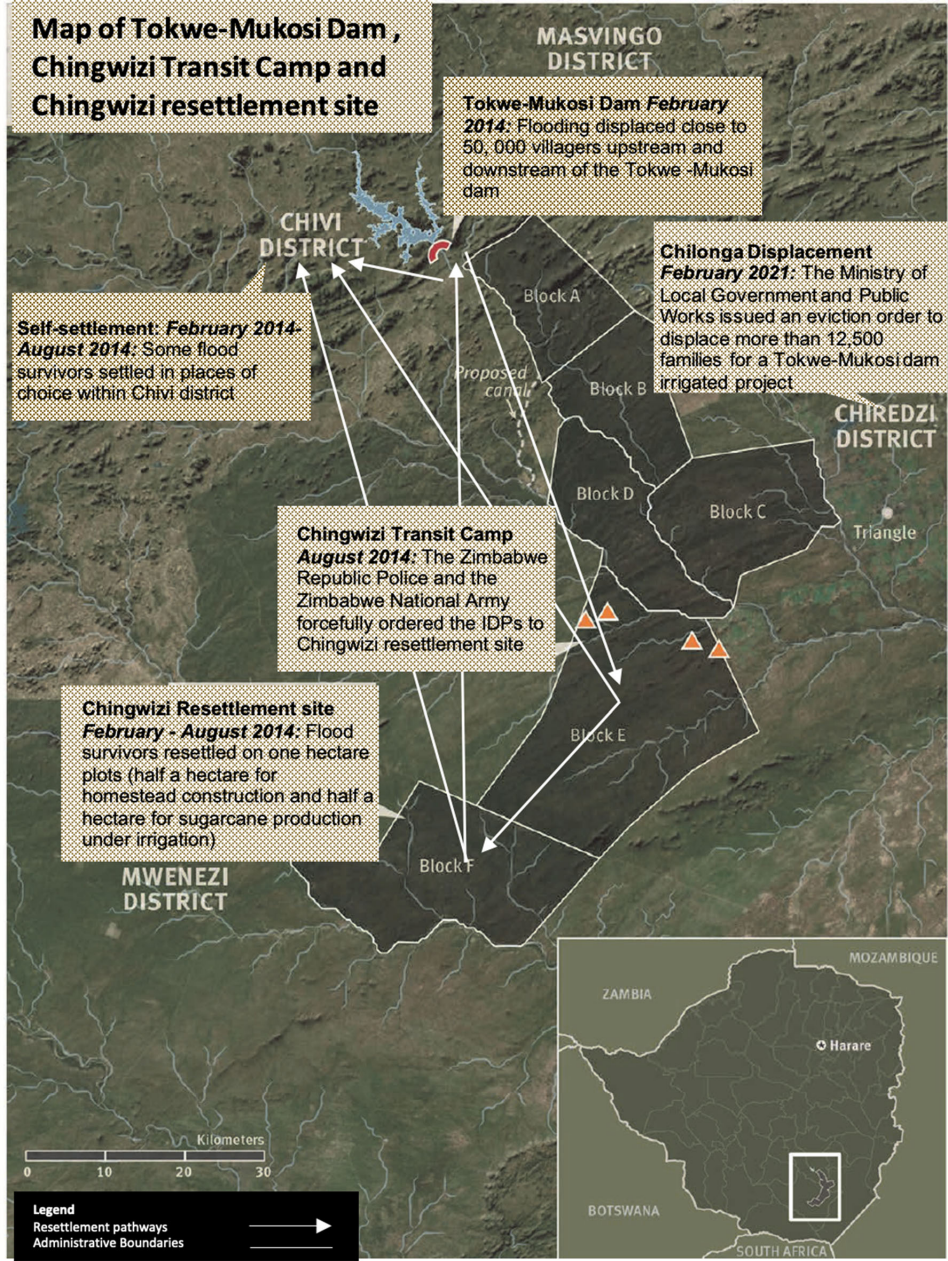
Tokwe-Mukosi Dam, Chingwizi transit camp and resettlement site ( adapted from Human Rights Watch, 2,015)

The SFDRR *supposedly* informs the approach to disaster management in Zimbabwe (Manyena, 2016). However, the archaic Civil Protection Act of 1989, which is still the chief law governing disaster risk reduction in the country, does not reflect elements of the SFDRR or its predecessor, the Hyogo Framework for Action 2005–2015, especially regarding substantially reducing risks for losses in lives, livelihood opportunities, and property (UNISDR, 2015). As Belle et al. (2017), Bongo et al. (2013), and Mhlanga et al. (2019) note, the legislation is more reactive than proactive. Our study is conducted in this context, to critically explore how the reactive policy stance affects resettlement during disaster-induced resettlement, focusing on the unprecedented disaster in Tokwe-Mukosi. The Tokwe-Mukosi dam is in the Masvingo Province, south of Zimbabwe. Chivi District, the origin of the Tokwe-Mukosi communities, receives an average annual rainfall of 400 mm typically, but between January and March 2014, received 850 mm of rainfall (Tarisayi, 2014). While some resettlement efforts were underway, the incessant rainfall breached the dam walls of the Tokwe-Mukosi dam inundating 5,793 families upstream and downstream (Mavhura et al., 2017). The government evacuated the households to the Chingwizi Transit Camp (CTC) and then to a subsequent resettlement area in Mwenezi District (Mukwashi, 2017), 170 km from the Tokwe-Mukosi dam site (Gumindoga et al., 2014) (Fig. 1).

We used an inductive approach to understand how the Tokwe-Mukosi community was displaced and resettled in Chingwizi, employing document reviews and narrative analysis (Johnston, 2014) to appreciate different ontologies for making sense of the disaster’s fallout. Focusing on how state actors assign particular notions of disaster risk reduction to the resettlement process, we recognise meaning-making as a product of different subject positionalities and experiences (Pope & Mays, 2020). Here we seek to build a politically sensitive narrative surrounding the resettlement processes. The first author conducted a field visit to the Tokwe-Mukosi and CTC in 2014, providing an opportunity to put questions to government authorities. The second author has been conducting in-depth ethnographic research in communities impacted by Cyclone Idai in 2019 in areas near the Zimbabwe-Mozambique border to understand their perspectives and experiences of displacement and resettlement, where references to the Tokwe-Mukosi resettlement were, due to its prominence, repeatedly invoked, alongside wider policy concerns and fears. We collected data between May 2020 and March 2021, through a document analysis method, focusing on literature concerning the Tokwe-Mukosi resettlement process and its representation, using multiple data sources comprising scholarly sources, non-governmental organisation (NGO) and government reports, conference proceedings, and newspaper articles to fill gaps and identify biases and inconsistencies within the data (Silverman, 2018), enabling verification. Focusing on the 2010 to 2021 period allowed for understanding the historical roots and injustices embedded in the resettlement processes.

We first conducted a systematic literature search on Web of Science and Scopus to obtain articles on Tokwe-Mukosi, refining the search terms using various combinations of: *Tokwe-Mukosi, flood, disaster, dam, displacement, transit-camp, holding camp, Nuanetsi, Chingwizi, ranch, resettlement and relocation*. After manually removing all duplicated articles, the process yielded only 15 articles. We then used snowball sampling to expand the literature search, searching for both academic and grey literature within the references of the identified articles. We selected articles which included the pre- and post-resettlement process of the Tokwe-Mukosi disaster, including the movement to CTC and the final resettlement in Chingwizi within the Nuanetsi Ranch, as well as the Tokwe-Mukosi dam construction period. Since we needed current information on actions that the government has taken based on the resettlement process, we included newspaper articles obtained from *The Herald* and *The Standard* from 2018–2020, using the same combination of search terms as used in the systematic search. The final data set consisted of 5 government reports, 7 NGO reports, 18 journal articles or book chapters and 25 newspaper articles. We used thematic analysis (Chandra & Shang, 2019) employing NVivo 12. To maintain a systematic analysis, we followed Creswell's (2009) six-step procedure to reiterate and reflect between the different stages and organise and describe the vast amounts of unstructured qualitative data in rich detail (Nowell et al., 2017), while considering all aspects of and potential biases in the data.

Keenly aware that documents cannot be treated as objective reflections of reality as they represent the views of those who write them, and are produced for diverse purposes, complementary analyses were derived from our research with disaster survivors in other locations–particularly in areas heavily impacted by Cyclone Idai, where discussion of what occurred in Tokwe-Mukosi was a frequent point of conversation. We also conducted an interview with personnel from the MLGPW, as well as a constitutional law expert, in addition to deriving insights from participating in webinar sessions together with displaced community members, civil society organisations and members of parliament with experiences of displacement in their constituencies.

## Tokwe-Mukosi’s resettlement processes

### Compensation and its failings

Between 2012 and December 2013, MLGPW resettled 600 of the 896 compensated households to Chisase and Masangula areas (Catholic Commission for Justice & Peace in Zimbabwe [CCJPZ], 2014; Chipangura et al., 2019). Initially, MLGPW planned to resettle the affected 6393 Tokwe-Mukosi households in three phases by October 2015, before the anticipated filling of the dam in December 2015 (Betera, 2014). This figure was 48% lower than the 1,247 households that were actually targeted for resettlement in that same period. Most of the resettlement processes were also reactive, as MLGPW only resettled the remaining 5793 households in the aftermath of the flood disaster (Mavhura, 2020), such that the delayed relocation and subsequent flooding resulted in the death of six people from drowning, loss of crops, livestock and property (Moyo, 2014 cited in Hove, 2016; Tarisayi, 2014). Reasons for stalled proactive resettlement processes included the central government’s lack of financial resources to compensate and transport the remaining households and refusal by the households to be relocated without full compensation from MFED (Betera, 2014; Mavhura et al., 2017) to enable the IDPs to re-establish their homes, initiate income-generating activities, restock livestock, and fulfil food, health and education needs (Zimbabwe Human Rights Commission [ZHRC], 2014).

While the MLGPW compensated the Tokwe-Mukosi IDPs using cash payments and land-for-land strategies, only the 896 households valuated before the flood disaster received a lump sum payment (Betera, 2014); the others only received funds in instalments, not fully paid until five years after the displacement. This left flood survivors unable to purchase suitable building material, replace lost assets or meet basic needs in the period between eviction and rebuilding. As a result, IDPs slept in the open and later in tents before they managed to build any housing infrastructure (Oxfam, 2014). In contrast to the more durable brick houses with zinc roofs (Mukwashi, 2019) built by those compensated early, households resettled after the flood received much less to cover immediate needs such as transport, food, education and health, among other unforeseeable costs, leaving little or no allocation for rebuilding houses. ZHRC (2014) and Mukwashi (2019) observed that households which received staggered compensation mostly built their houses from poles and mud, structurally vulnerable to windstorms. In 2019 windstorms destroyed more than 100 houses, condemning them back to tents provided by NGOs and the government (Maponga, 2019a).

The 600 households resettled in Chisase and Masangula received the planned land-for-land compensation (Chipangura et al., 2019). In contrast, households in Chingwizi only received half a hectare–designated by the government for sugarcane production under irrigation, and another half for constructing a homestead (Human Rights Watch [HRW], 2015). Since the size of the land was smaller than what the families previously owned in Tokwe-Mukosi, the smallholder farmers lost land for growing subsistence maize crops and small grains (ZHRC, 2014), individual and community gardening (Mutangi & Mutari, 2014) and livestock production (Betera, 2014). The HRW (2015: 22) recorded an interview where one former village leader expressed discontent with the unilateral decision, saying: “…we are now being forced to be sugarcane farmers. We have no previous experience in sugarcane farming; neither do we have an interest in it.” In Chingwizi, privately owned land surrounded the households, leaving IDPs with no land for livestock production. While households claimed that their livestock died due to lack of pastures (Maponga, 2019b), compensation for these losses was denied, with the government stating that the law explicitly restricts compensation to improvements and crops made at the place of origin (Vengesai & Schmidt, 2018).

Since the MLGPW resettled the communities in new village patterns, social networks were upset, with losses in Chingwizi including disrupting extended family members, church members, friends and village neighbours (Atahar, 2014; Mutangi & Mutari, 2014; Roca & Villares, 2012). These social networks carried with them intangible opportunities such as microfinance investment, locally known as *mukando*, based on longstanding relationships of mutual trust (Mutangi & Mutari, 2014). Previous state-orchestrated, post-millennium displacements demonstrate that the Zimbabwean government has no history of preserving social networks when resettling IDPs. Potts (2008) notes that during the 2005 Operation Murambatsvina government evictions, the MLGPW trucks offloaded some families near their communal areas, while it randomly resettled others in former farmlands without any spatial configuration considered.

Income losses included the loss of trade opportunities. More than 24 informal traders from 12 affected villages lost all their stock during the floods, as well as loss of customers (Tarisayi, 2014). The informal traders had mostly sold clothing, foodstuffs, and electrical goods obtained from South Africa (Betera, 2014; Mavhura, 2020); others locally traded in fish, fruits and vegetables grown in the nearby Runde catchment area river sources (Mutangi & Mutari, 2014). After the disaster, the majority of traders in Chingwizi had to survive by selling cheap products made from natural resources such as reed mats, baskets and grass brooms (Mukwashi, 2019). However, the ZHRC (2014) noted that the business was unlucrative because similar products flooded the market, and the clients, usually fellow IDPs, could not purchase the products as they lacked stable income sources. Additionally, the lack of property and business insurance (Mavhura, 2020), compounded by a lack of collateral assets to secure financial loans, reduced IDPs’ access to the financial opportunities required for them to recover. Without security against income shortfalls and alternative profitable livelihood opportunities, displaced communities remained perpetually deprived (Deeyah & Akujuru, 2017).

Importantly, the government only compensated immovable property while overlooking essential social infrastructure in the camp and new site. Displaced households still live in areas with inadequate health, water and sanitation, education, community centres, transport and other essential infrastructure (Mavhura, 2020). In Chingwizi, the MLGPW built five schools, two clinics and 63 boreholes only after the arrival of the IDPs to the resettlement sites (HRW, 2015), leaving a considerable shortfall from the initially planned development. Previous dam related displacements in the country show that once the state relocates IDPs, its support dwindles, as documented by Makururu et al. (2018) with respect to the state’s withdrawal from displaced communities in Eastern Zimbabwe after the Osborne dam construction. Almost 30 years after the dam’s completion, the resettlement area still lacks physical infrastructure like irrigation pipes, canals, schools and clinics (ibid). In addition to boosting the income-generating activities of the IDPs, Kalin (2014) notes that upgrading infrastructure is also essential to reduce tensions between IDPs and host communities.

Critical narratives also point to how the government ignored compensation of cultural and spiritual losses such as religious sites and gravesites (Mutangi & Mutari, 2014). Mwandayi (2011) observed that graves are an integral part of the traditional rituals of rural households in Chivi district, which include rain making ceremonies at the community level and protection at an individual level. The compensation process fractured the unacknowledged spiritual connection to religious sites that provide meaning to the daily lives of ordinary people. Hollenbach (2014) underscores the value of religion and spirituality in helping people to cope with trauma, reduce anxiety, gain social support and commune with the sacred. While MLGPW has previously assisted in relocation of the graves (Manyanhaire et al., 2007), the speed with which the Tokwe-Mukosi disaster occurred provided no time for exhumations to take place. Even though alternative remedies could have been sought, such as providing space for religious sites in the resettlement area, an interviewee from the MLGPW noted that the ministry could not provide alternative remedies due to lack of funds. López-Carr and Marter-Kenyon (2015) also suggest the relocation of an entire village together to preserve cultural and social network bonds, which MLGPW overlooked in Chingwizi.

The compensation method used by MLGPW, which focuses on immovable assets and any other damages the affected households incur during the relocation process (Vengesai & Schmidt, 2018) also ignores individual-level losses, such as place-based knowledge from longstanding relationship with animals, land, forests, rivers, air and the sky, which takes years to establish. In Chivi, this knowledge contributes to decision-making on animal and human health, natural resource management and agriculture (Maunganidze, 2016). Another type of uncompensated individual level loss includes significant leadership roles which vanished after the displacement. Thirteen village leaders from Chekai, Jahwa, Zifunzi, Mharadzano, Chikandigwa, Nemauzhe, Tagwirei, Ndove, Matandandizvo, Mashenjere, Nongera, Chikosi, and Neruvanga (Bwerinofa & Kudzai-Chiweshe, 2017), and two chiefs (Neruvanga and Nemauzhe) lost their leadership roles (Betera, 2014). These losses, while occurring at individual-level, transcend individual boundaries by becoming intergenerational losses of identity and knowledge.

While skills development and training plays a vital role in long-term resettlement planning by allowing IDPs to diversify their livelihoods (Mukwashi, 2017), livelihood opportunities in most rural-to-rural resettlement tend to focus only on the agricultural sector (Tadgell et al., 2018) despite the fact that agricultural livelihoods might be under threat in the new location (Al Atahar, 2014). This observation resonated with the Chingwizi agricultural focus, with the assumption that the Ministry of Agriculture, Water, and Rural Resettlement (MAWRR) would train the community in sugarcane production. Moreover, without irrigation, Chingwizi faced more severe water shortages compared to Tokwe-Mukosi, which existed near water sources (Chazireni & Chigonda, 2018). Usamah and Haynes (2012) underscore the importance for governments to consider community preferences complemented with both new and old skills training and long-term viability. Based on the activities that the Chingwizi IDPs carried out before and after the resettlement, alternative skills to agriculture that could have been strengthened by the government before resettlement include, but are not limited to, construction, selling in markets and microfinance skills.

The pre- and post-flood resettlement processes, which we outline in Table 1, illustrate key developments in the construction of the Tokwe-Mukosi dam and related plans regarding the sugar plantation, electricity supply, fisheries, and recreation and tourism facilities aimed at growing the provincial economy. Embedded in this timeline are also the advocacies by IDPs and political elites dissatisfied by the government resettlement processes. In 2014, then Masvingo Provincial Affairs Minister Kudakwashe Bhasikiti declared that IDPs had adequate relief supplies (The Financial Gazette, 2014), despite widespread shortages (Hove, 2016). However, after dismissal from his ministerial post, Bhasikiti, moved a motion in parliament calling on the government to complete the resettlement and irrigation scheme immediately and provide full compensation to the IDPs (The Financial Gazette, 2015b). In 2015, Bhasikiti’s successor, Masvingo Provincial Affairs Minister, Shuvai Mahofa vowed to resettle the IDPs in a better place as they were currently ‘living in a place fit for animals’ (The Financial Gazette, 2015a). Regardless of the politicians’ admission that conditions in the resettlement site were unsuitable, in 2014, the police arrested the Chingwizi camp committee leadership, including the chairperson, Mike Mudyanembwa, for protesting the conditions (HRW, 2015), with the courts sentencing him to a five-year jail term in 2015. Subsequently, Minister Bhasikiti accused the Chingwizi camp committee leaders of influencing the flood survivors to reject resettlement without compensation, which he claimed, the majority of flood survivors wanted (ibid).

**Table 1:** Main resettlement processes between 2011–2020

Year	2011	2012	2013	2014	2015	2016	2017	2018	2019
Initial Plan before the flood disaster	Salini Impregilo resumes dam construction	MLGPW valuation of property and initial compensation	MLGPW finalises compensation of IDPs, drilling of 100 boreholes, construction of five schools, one clinic 12 dip tanks	MLGPW completes Phase 2 resettlement of 1 878 households by October	MLGPW completes Phase 3 resettlement of 3 268 households by October	Government’s initiates 25 000-hectare sugar plantation, 6–15 MW electricity supply, fisheries and recreation facilities			
		MLGPW Initiate resettlement	MLGPW completes Phase 1 resettlement of 1 247 households by October		Anticipated completion and filling of the dam by December				
Actual events before, during and after the flood disaster	Salini Impregilo resumes dam construction	MLGPW compensate 896 households @MLGPW phase 1 resettlement of 600 households to Chisase and Masangula by December		Presidential declaration of flood disaster and evacuation of 5, 793 households to Chingwizi Transit Camp in February			Dam project completed	Partial compensation of IDPs by MLGPW and MFED	Final compensation of IDPs by MLGPW and MFED@Once off relief distribution by humanitarian agencies to 70 Chingwizi households affected by a January 2019 storm
				Voluntary resettlement of IDPs to Chingwizi resettlement site					
				Provision of relief by humanitarian organisation’s to IDPs until August					
				Declaration by Masvingo Provincial Affairs Minister Kudakwashe Bhasikiti that IDPs have adequate relief supplies despite widespread shortages	Demand by Kudakwashe Bhasikiti for government’s completion of the resettlement and irrigation scheme, and full compensation of the IDPs				
				Police arrest of the Chingwizi camp committee leadership for protesting against the inhuman conditions within CTC	Masvingo Provincial Affairs Minister Shuvai Mahofa vows to resettle the IDPs in a better place				
				Violent closure of the camp and forced resettlement in Chingwizi in early August by army and police	Chingwizi camp committee chairperson, Mike Mudyanembwa, jailed				
				Chingwizi camp committee leaders accused by Bhasikiti of inciting flood survivors to reject resettlement without compensation					
				MLGPW construction of two clinics, five schools, 63 boreholes					

## State power and the resettlement processes

### Diverse forms of exercising power in the resettlement processes

The nexus between state power and the Tokwe-Mukosi resettlement processes started from the conception of the dam project and has no end in sight as the IDPs still live in limbo. Our analysis shows that the construction of the dam and the subsequent resettlement objectives depict clear state-driven capitalist values. The intended 25,000-hectare sugarcane irrigation project in the province involving the IDPs (Betera, 2014) alters the agricultural livelihoods of the households from being subsistence farmers to commercial farmers, constituting a cultivation of powerless and exploitable neoliberal subjects who neither have control over what to grow nor market forces. Additionally, the dam-related recreation and tourism facilities meant to grow the provincial economy benefit only the few elites with sufficient capital to invest in the hospitality industry (Mukwashi, 2017), with little benefit to the IDPs’ quality of life. Central government, through MFED, demonstrated its priorities after the disaster when it focused on completion of the dam while neglecting IDPs’ compensation (Government of Zimbabwe [GoZ], 2014). The processes of reducing disaster risk for the Tokwe-Mukosi community created new patterns of winners and losers reflected in the resettlement of the IDPs with capitalist motives of enriching the state’s elite and forcing IDPs into a market economy. IDPs rather than benefitting from development now endure hegemonic ideals of disconnection to place, destruction of social networks and a drive towards commercial-oriented agricultural production.

The state used its monopoly of power, exercised through MFED, MLGPW, ZRP, and ZNA to decide what was desirable to reduce disaster risk. The purpose and siting of the dam project was the state’s conception (Mukwashi, 2017). MFED controlled the value of compensation and payment terms, while MLGPW advanced a narrative that perceived IDPs’ demands for compensation as improper (Mavhura, 2020). ZRP and ZNA controlled access to humanitarian resources in the camp and were instrumental in forcefully evicting IDPs from the camp (Hove, 2016). MLGPW determined the location of the new settlement, including the settlement patterns and land use in the new site (Mukwashi, 2017). MLGPW excluded traditional authorities from the resettlement processes and abolished their authority in the new site (Mutangi & Mutari, 2014; Tarisayi, 2018). Since the dam completion in 2017, IDPs are yet to benefit from the promised dam projects. As argued by Ingalls and Stedman (2016), hegemonic application of social power results in privileging the interests of some actors over others, which creates distributional inequalities. We contend that the inequality and accumulation of power in the state’s elite is increasing rather than reducing vulnerability. As such, we argue that it is essential for scholars to focus clearly on power relations, avoiding presumptions about homogeneity or equal ability of individuals, communities and nations to coping with challenges of displacement.

There were three main ways in which the IDPs were resettled, attributable to different circumstances and preferences. The first resettlement way was without direct physical force. MLGPW resettled households either directly to their permanent sites from Tokwe-Mukosi or after briefly staying in the camp before ‘volunteering’ to move to the permanent site. However, Oxfam observed that: ‘…some households had to carry their belongings from the road to their plots walking approximately 4 km into the bush looking for their pegs. The plots were not cleared, neither were they habitable. Each family was allocated tarpaulin plastic sheeting without timber…’ (Oxfam, 2014: 6). Voluntary resettlement of encamped IDPs continued until the forced closure of the camp. During the first author’s visit in April 2014, the Mwenezi District Administrator explained that the government was offering free transport to families who volunteered to resettle to permanent sites. That same month, the then-MLGPW minister, Ignatius Chombo, threatened to stop providing relief to the IDPs, if they declined to move out of the camp to the one-hectare plots without compensation; only 400 out of over 18,000 yielded to the pressure (Hove, 2016). Here the state apparatus used economic force to enforce the will of elites.

The second method of resettlement involved the police and army’s use of outright physical force. After the majority of the IDPs refused to move out of the camp without compensation, the government relocated the clinic to the final resettlement site in early August (Hove, 2016). The IDPs protested violently, disarming anti-riot police and burning two ZRP vehicles in the process (HRW, 2015). In mid-August, ZRP and armed ZNA soldiers arrested over 300 IDPs, destroyed the temporary shelters, and ordered everyone to the new resettlement site (Hove, 2016). Violence was further augmented by the fact that the Nuanetsi ranch where Chingwizi is situated had disputed ownership, claimed by Development Trust of Zimbabwe (DTZ), a company aligned to the ruling Zimbabwe African National Union-Patriotic Front (ZANU-PF) party (Hove, 2016). Reviewed literature shows conflicts even before the 2014 resettlement. Mujere and Dombo (2011) report that in 2010, DTZ obtained a court order to evict twenty-five households and their 12,000 cattle from the ranch during the country’s 2000–2003 Fast Track Land Reform Programme. These existing tensions continue to threaten to evolve into another displacement for the IDPs leading to another disconnection of place, people and livelihoods. In 2019, tensions over grazing land and water sources led the Minister of State for Masvingo Provincial Affairs, Ezra Chadzamira, to plead for dialogue saying: “You can share the available resources, especially water…it is very disturbing to note that families here have livestock which has no access to grazing pastures because grazing lands have been fenced off” (*The *Herald, 2019). Mavhura (2020) also recently reported tribal tensions between the resettled Karanga community and the hosting Shangani community over the sharing of arable land and pastures. Apart from potential displacement, the prohibition to own cattle by the MLGPW, and conflicts with the host community over livestock production disconnected the IDPs from cattle rearing, one of the only thriving livelihoods in the district (Chiruvu et al., 2017).

The third type of resettlement was self-settlement outside Chingwizi after the violence. The IDPs either moved to other places of choice within Chivi (Zikhali, 2018) or back to the original resettlement site (Hove, 2016). Zikhali (2018) notes that IDPs who moved to various parts of Chivi District were in search of autonomy from government control, agricultural land for livestock and crop farming. IDPs who moved back to Tokwe-Mukosi still had habitable homesteads, unaffected by the floods (ZHRC, 2014), despite the risk of future flooding. Since most households remained in Chingwizi, the returnees also risked limited physical connection to families and neighbours in Tokwe-Mukosi. The government, however, vowed to evict those who had resettled in the dam basin (Hove, 2016). The self-settlement reflects the preference by many IDPs to pursue self-defined vulnerability reduction characterised by autonomy and preference for settlement and livelihood opportunities outside the limits of state-defined vulnerability reduction.

### Unsuitable settlement (Encampment) at Chingwizi: a centre of state-perpetuated violence

One of the government’s justifications for setting up the camp was creating a central logistical coordination for meeting IDPs’ humanitarian needs (Betera, 2014). However, Samu and Kentel (2018) point out that the government met the humanitarian needs of IDPs in the country’s previous emergencies without encamping the victims, noting that in 2000, aid agencies provided humanitarian aid to more than 500,000 Cyclone Eline survivors in the Limpopo and the Save River basin, without encamping them. Therefore, using this argument for encampment seems disingenuous.

Moreover, World Food Programme and other humanitarian agencies barely met the critical food and non-food requirements (Betera, 2014) in the Chingwizi camp. One flood survivor interviewed by the CCJPZ (2014) summed up the challenges in the camp by saying:

We have inadequate food and shelter. Sanitary, ablution and healthcare facilities are scarce. We do not have boreholes or any reliable clean source of water. The tents that were donated as a form of shelter are few and therefore crowded…We do not have schools and our children have been out of school for long…We are not sure when the situation will improve (CCJP, 2014: 9).

Even though the central government set up the camp supposedly to provide physical security to the large number of IDPs, it became a centre of state perpetrated violence and abuse (Hove, 2016). Madzokere (2018) notes that the police failed to adequately prevent the host community from stealing donated goods as the outsiders masqueraded as IDPs. Betera (2014) also reports that ZRP officers and state officials stole the supplies the community expected them to protect and equitably distribute. While the MLGPW assumed greater control of aid distribution by directing that all aid pass through the Minister of State for Masvingo Provincial Affairs, Hove (2016) argues that authorities diverted donated goods to sell these in the surrounding towns of Triangle and Chiredzi leading to significant losses of aid meant for IDPs. Typical of camps, the police enforced restricted movement in and out of the camp (HRW, 2015), restricting livelihood opportunities for IDPs in employment, pastures and firewood (Bakewell, 2014). Towards the closure of the camp, the police and army, through the MLGPW directive, denied and limited food and water, blocked toilets and closed the school and the clinic to force the IDPs out of the camp (Hove, 2016). The camp was, therefore, a centre of wielding power and control instead of being a haven for providing much-needed protection and personal security so that IDPs could swiftly revert to normal life.

Moreover, there was an increase in transactional sex in exchange for humanitarian aid controlled by male police officers. One survivor interviewed by the CCJPZ (2014) confirmed this view saying: “… vulnerable groups such as women and children have sacrificed themselves to access the few donations. Prostitution in exchange for humanitarian aid has become common.” Sexually transmitted diseases increased, and about 100 teenage girls between the ages of ten and twelve fell pregnant and dropped out of school (Hove, 2016). The camps, instead of being sanctuaries of safety, increased the risk of exploitation and abuse.

While the clinic in the camp facilitated access to health services, the conditions at the camp exacerbated the vulnerability of the IDPs to various diseases. Sanitation coverage was inadequate and on arrival at the camp, IDPs had no access to safe drinking water. With the nearest safe water source, a borehole, 30 km away, the IDPs relied on uncovered stagnant water pools for domestic water use (Oxfam, 2014). The inadequate water and sanitation conditions increased diarrhoeal disease, with 60 cases recorded in the health camp facility in March 2014 alone (United Nations Development Programme, 2017). In total, seven fatalities occurred in the camp, compared to the six who drowned during the flood (Hove, 2016). The camp registered increased malaria and tuberculosis cases, there was no ambulance to transport the sick, and the clinic structure, a makeshift tent, was inadequate for the provision of quality care (United Nations Office for the Coordination of Humanitarian Affairs [UNOCHA], 2014). Results from a March 2014 Ministry of Health and Child Care survey in Mwenezi district on the nutritional status of children under the age of five revealed that 2% of children in Chingwizi received a minimum acceptable diet compared to 68% of children living outside the camp (Chiruvu et al., 2017). The health and nutrition situation in the camp was inconsistent with assisting the IDPs towards recovery.

An estimated 800 primary and 500 secondary school pupils missed school in the early days at the camp (Madzokere, 2018). During the first author’s visit to the camp in April of 2014, it was learned that only one teacher was available to teach all the primary and secondary pupils under one makeshift tent. Vulnerable to various weather elements such as rain and cold, pupils sat on the ground due to furniture shortages, in addition to lack of toys, books, water and sanitation facilities for the school (UNOCHA, 2014). Some 400 pupils dropped out of school to fend either for themselves or their families (Hove, 2016), further exacerbating future opportunities.

Mujere and Dombo (2011) highlight that cattle production is a significant enterprise in the Chingwizi area. Apart from their consumption, cattle are an essential facet of Zimbabwe’s rural economy–used for transportation of inputs and produce, firewood and water; capital growth and storage, through herd growth; tillage; and cultural ceremonies such as paying bride prices (Matope et al., 2020). Despite the importance of livestock production in Chingwizi, there is no record of training the community on animal husbandry to reconnect the IDPs to their farming activities; many cattle acquired diseases (UNOCHA, 2014), and, as noted above, there was no compensation for those who lost livestock during the flood or transportation. Tarisayi (2014) established that the IDPs lost goats, sheep and cattle during the floods, and Mutangi and Mutari (2014) noted that others died on their way to the transit camp. The MAWRR thus missed critical livestock support opportunities that could have improved the lives of the IDPs in Chingwizi.

#### Unfulfilled promises

Three main policy reforms were flagged for action but remain unfulfilled, related, respectively to legislative reform, compensation guidance and management of camps**.** In a 2014 report produced by the Directorate of Civil Protection assessing the management of the Tokwe-Mukosi disaster (Betera, 2014), a review was recommended of the Civil Protection Act of 1989, the principal law governing disaster risk reduction in the country. Mavhura (2016: 611) notes that the Act uses ‘a command-and-control model derived from a militaristic system.’ Moreover, Sect. 29 to 37 of the Act establishes funding at a national level through the National Civil Protection Fund (GoZ, 1989), without establishing such funding at the local level where the disasters occur. The local authorities barely receive funding from the central government before disasters occur (Mavhura, 2016), such that the central government only avails resources after a disaster, tightly controlling the funds needed for community disaster risk reduction. Parliament failed to amend the legislative framework several times since 2003 (Mavhura, 2016), despite the view by Capacity for Disaster Reduction Initiative (CADRI, 2017) that the proposed 2011 bill was antagonistic to the SFDRR by ignoring long-term needs. While an interviewed legal expert argued that such laws were unjust and hence unconstitutional and undemocratic, two interviewed members of parliament blamed the affected communities themselves for not challenging the injustices. Our analysis conforms with that of Cretney (2017), namely that the promotion of neoliberal policies to boost economic growth and enforce political legitimacy undermine resilience to forced displacement related to climate-induced and other disasters. We contend that the state’s resistance to amending the legislative framework, based on this desire to maintain hegemonic opportunities for control and authority, is counterproductive to social equity.

Revised compensation guidelines, also recommended by the report, are yet to be developed or made public and the complex interplay of state institutions, politicians, and non-state actors regarding responsibility for forced displacements remains unaddressed. Notably, in this regard, the district council officers with whom the second author met in other parts of the country in 2019, acknowledge that political considerations were behind their reluctance to endorse the recommendations in a NGO report that called for fundamental changes in resettlement and compensation policy. Deeyah and Akujuru (2017) conceptualise compensation as fault finding, where the guilty party recompenses the victim. If state institutions adopt this same conceptualisation of compensation, then victims of disaster-induced displacement might stand to lose amidst blame shifting on responsibility to act before, during and after a disaster. For instance, in the Muzarabani and Mbire floods, survivors blamed their Rural District Council for neglecting them during the disaster and the district councillors blamed the survivors for settling in flood-prone areas (Mucherera & Mavhura, 2020; Ncube-Phiri et al., 2014). While compensation guidelines might be a lifeline for affected rural households known to live without insurance schemes (Mucherera & Mavhura, 2020), the state’s reluctance to engage in developing new guidelines cannot be seen as mere oversight. Such an effort would raise the question of whose knowledge and values matter when considering what to compensate and how much it is worth.

In this vein, it is telling that various interviewees in Chimanimani District in early 2020 articulated diverse views on why a private company–Econet–ultimately did not follow through with its publicly-announced commitment in 2019 to fund a resettlement housing programme for Cyclone Idai IDPs. Some narratives indicated that it was powerful national government elites (not government officials in the district) who rejected the proposal fearing that the model might be “too good.” One critique was that political elites did not want to create a scenario where Econet (owned by a businessman with rumoured political aspirations) might claim too much of the credit. Another narrative was that officials feared creating a precedent that housing structure programs for resettlement communities would be better than for others; yet another narrative was that “Econet was lying and did not have a budget committed with the promises.

In a more recent 2021 case, the Chilonga community in Chiredzi district, south east of Chivi district, is on the verge of being evicted from their communal lands without compensation. An interviewee from Zimbabwe Coalition on Debt and Development decried the combined business community and state collusion in oppressing rural communities, noting that the land in Chilonga was earmarked for lucerne grass production for Dendairy, a private dairy company in Zimbabwe. The grass will be irrigated using water from the Tokwe-Mukosi dam, despite Chilonga being further downstream. One interviewee from the Masvingo Centre for Research and Advocacy highlighted that MLGPW took advantage of the absence of a compensation framework to publish Statutory Instrument 50 of 2021 followed by Statutory instrument 63A of 2021 to evict at least 12, 500 indigenous Shangani households from their Chilonga communal land. The legal expert interviewee emphasised that even though the MLGPW evoked the Communal Land Act and crafted the statutory instruments, the move was unconstitutional because Sect. 74 of the constitution guarantees every Zimbabwean the right to a home–the Chilonga community were being displaced from a place they call home. In addition, the eviction lacked authorisation from a court of law and failed to consider alternative remedies before the ensuing eviction.

The Chilonga case points to the dam’s far reaching effects in adding to the constellation of already existing injustices. The arbitrary eviction for capitalist gains and lack of compensation without alternative land and infrastructure provision emphasize the unrelenting use of state power under the guise of disaster risk reduction. Additionally, the Chilonga case echoes the Tokwe-Mukosi displacement saga, underscoring how the failure to meaningfully apply the lessons from Tokwe-Mukosi are resulting in repetitions of resettlement injustices.

Regarding encampment practices, despite the myriad of challenges observed in CTC, the central government still adopted encampment in response to the Cyclone Dineo and Idai disasters, the two significant disasters after the Tokwe-Mukosi. Similar to CTC, in Tsholotsho, during Cyclone Dineo, IDPs experienced inadequate food security, water and sanitation, slept communally due to shortage of tents in the camp which deprived the IDPs of privacy and increased the risk of communicable diseases (Department of Civil Protection, 2017). Significantly, 953 (3%) of Cyclone Idai IDPs in Chimanimani are encamped and experiencing these similar challenges, almost 17 months after the disaster (UNOCHA, 2020). Given the drawbacks of large encamped vulnerable populations and the ongoing COVID-19 (Coronavirus) pandemic (Kassem & Jaafar, 2020), the camp setup is widely seen as a health timebomb. However, since camps are spaces for consolidation of power through securitisation and resource control (Jacobs & Kyamusugulwa, 2018), other alternatives can be a threat to the established power in camps.

The choice by 97% of the Cyclone Idai IDPs to stay with the host community consisting of relatives and friends (UNOCHA, 2020), highlights a form of resistance to widespread control and underlying injustices in camp setups. Indeed, the second author of this study found multiple people affected by Cyclone Idai, in Chimanimani, who referenced the Tokwe-Mukosi saga when discussing their own fears of being “forgotten” and “ignored” if they just waited in tents in camps. That camps are also places where neoliberal corporate and economic elites manipulate shocked populations was not lost on the Chimanimani flood survivors. Our observation concurs with that of Bhagat (2020) who also notes that protracted encampment supports neoliberal tendencies through increased authoritarian surveillance and institution-led survival strategies of self-reliance such as microfinance and entrepreneurship. While some literature frames IDPs as passive victims (Priorelli, 2021), the challenge to encampment in Chimanimani illustrates resistance to power inequalities tied to superficial efforts of poverty alleviation and a potential opportunity for alternatives to encampment. Unfortunately, the host communities in Chimanimani now bear the burden of food and shelter provision for the IDPs without external support. Many of the NGOs that were ready to provide resettlement support in the year and half after Cyclone Idai were constricted in their efforts, at times because they were told that Econet was going to do it (before the Econet project plan fell through), resulting in opportunities lost. Similarly, IDPs in Tsholotsho returned to unrepaired dilapidated homes devoid of food and other welfare needs (Dube et al., 2018; UNOCHA, 2020), while those in Chingwizi settled on bare ground without assistance. This practice is, however, consistent with neoliberal norms and values of dismantling state welfare for IDPs (Bhagat, 2020), demonstrating an unrestrained application of power against the values of disaster risk reduction.

## Conclusion

The pre- and post-flood induced resettlement processes in the Tokwe-Mukosi disaster illustrated how state power shaped the form of community vulnerability during forced resettlement processes. Our research draws attention to how broader systems of injustices (Boonstra, 2016; Fabinyi et al., 2014; Ingalls & Stedman, 2016; Taylor, 2015) increase vulnerability, with Zimbabwe’s case study showing how capitalist motives and values impoverished its powerless subjects rather than building their resilience. As Newman (2010) argues, the state can act as a tool to perpetuate capitalism as well as a locus of power to protect its interests. Here the Zimbabwean state, through its institutions (MFED, MLGPW, ZRP and ZNA), used the disaster to promote a new vision of security in an uncertain future (Aradau, 2014), while expanding capitalist accumulation processes at the expense of communities in need of its protection.

By mapping the Tokwe-Mukosi pre- and post-flood period, we illustrated the complexity of resettlement processes, showing how the flood disaster catalysed and threw the planned processes into disarray when unanticipated torrential rainfall unexpectedly filled the dam. IDPs’ vulnerability increased throughout the resettlement processes, with the MLGPW, MFED, ZRP and ZNA using their power to create losses in land sizes, compensation, social networks, livelihood opportunities, social infrastructure, cultural and religious sites, place-based knowledge, and individual-level losses and reducing the ability of the IDPs to restore their livelihoods in what, for them would be fair, equitable and inclusive ways.

Regarding how state power contributes to the vulnerability of the Tokwe-Mukosi communities’ forced resettlement, our findings revealed multi-faceted state power relationships during forced resettlement processes. First, the central government conceived the dam project and unilaterally decided how the displaced communities would return to normal functioning by dictating where the IDPs would resettle, the livelihood opportunities on which they would embark, and the resettlement pattern, which affected the IDPs’ connection to livelihoods, people and the new place. During the flood disaster, the state decided the resettlement trajectory of the IDPs, which began by MLGPW randomly resettling them in the CTC, tearing connections between people. In the camp, the state controlled access to food, water, sanitation, shelter, health and education needs for the IDPs. Instead of meeting the physical security needs of the community, the courts convicted camp committee leaders while the MLGPW, police and army ended up violently evicting the IDPs from the camp without compensation, thereby affecting the IDPs’ ability to revert to normal life. Shaping the level of vulnerability reduction by controlling IDPs’ connections to place, people and livelihoods, state institution actions promoted conditions that reproduced and increased poverty.

Ignoring the main lessons that could have been acted upon in the aftermath of the Tokwe-Mukosi resettlement process, the parliament, first, failed to review the militarised Civil Protection Act, which we interpret as a way of maintaining grip on a status quo that provides hegemonic opportunities for control and authority. Second, the central government is yet to craft the guidelines for compensating IDPs, raising questions about whose knowledge and values are being used to decide what to compensate and to what value. Third, the central government is yet to review its encampment practices, which may be maintained to retain neoliberal norms and values of authoritarian surveillance and consolidation of power. Ultimately, the resettlement processes point to the critical need for theorising varied displacement types and experiences, linking protracted struggles with power dynamics that span multiple scales.
